# Homogeneous catalytic reduction of polyoxometalate by hydrogen gas with a hydrogenase model complex†

**DOI:** 10.1039/c9ra04396a

**Published:** 2019-06-21

**Authors:** Takuo Minato, Takahiro Matsumoto, Seiji Ogo

**Affiliations:** Department of Chemistry and Biochemistry, Graduate School of Engineering, Kyushu University 744 Moto-oka, Nishi-ku Fukuoka 819-0395 Japan minato.takuo.219@m.kyushu-u.ac.jp ogo.seiji.872@m.kyushu-u.ac.jp +81-92-802-2823 +81-92-802-2818; International Institute for Carbon-Neutral Energy Research (WPI-I2CNER), Kyushu University 744 Moto-oka, Nishi-ku Fukuoka 819-0395 Japan; Centre for Small Molecule Energy, Kyushu University 744 Moto-oka, Nishi-ku Fukuoka 819-0395 Japan

## Abstract

The homogeneous catalytic reduction of a polyoxometalate (POM) by hydrogen gas in aqueous media was investigated for the first time by using a [NiRu] hydrogenase model complex (I) under very mild conditions. By bubbling hydrogen gas into the buffer solution containing I and the Dawson-type POM (II_ox_), the color of the solution turned from pale yellow to dark blue, suggesting the reduction of II_ox_. The catalytic and kinetic studies revealed that I acted as an efficient catalyst to yield one-electron-reduced Dawson-type POM (II_red_) with a low energy barrier for activating dihydrogen and reducing II_ox_*via* a hydride complex of I. The process for the one-electron reduction of II_ox_ was confirmed by UV-vis spectroscopy, controlled potential electrolysis, and X-ray photoelectron spectroscopy. POM II_red_ could stably store protons and electrons and release them by addition of oxidants, demonstrating that POMs acted as redox active mediators for transporting protons and electrons from hydrogen gas to acceptors. The recycle study showed that II_ox_ and II_red_ could be reduced and oxidized by hydrogen and oxygen gases, respectively, at least five times with >99% yield of reduced species, showing a durable system for extracting protons and electrons from hydrogen gas.

## Abstract 2

Homogeneous catalytic reduction of polyoxometalates by hydrogen gas with a hydrogenase model complex was investigated for the first time.

## Graphical abstract

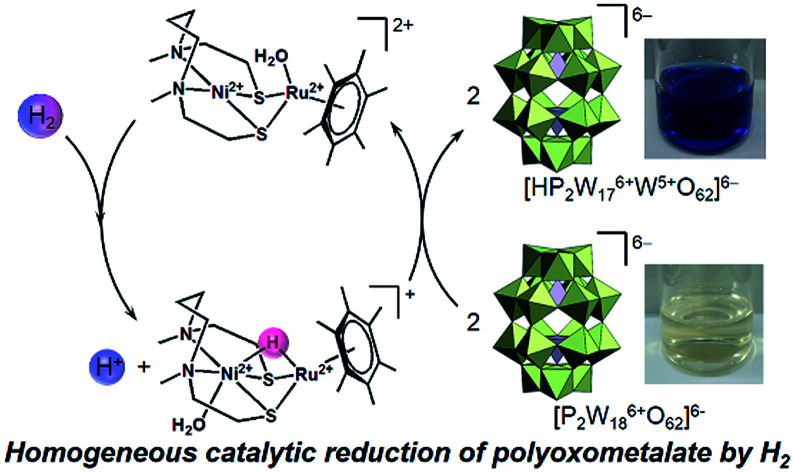

## Introduction

Polyoxometalates (POMs) are a class of anionic molecular metal oxide clusters that exhibit various unique chemical and physical properties.^1^ The redox properties of addenda atoms in POMs (W^6+^, Mo^6+^, V^5+^, *etc.*) have attracted considerable research attention because their highly stable redox states, which are based on the robust POM framework and the ability to delocalize electrons and protons on the anion, enable the exploitation of energy storage materials and redox catalysts, including electrocatalysts and photocatalysts.^2^ For example, Cronin *et al.* has recently reported that a Dawson-type POM can be electrochemically reduced up to 18-electrons per molecule, which can be utilized as a high-performance redox flow battery electrolyte and a mediator in an electrolytic cell for on-demand dihydrogen generation.^2*a*^ Although the reduction of POMs is a key step to store energy or to activate/regenerate catalysts, the reduction of POMs by hydrogen gas has been hardly investigated mainly because the reduction of POMs has been performed by using a (super)stoichiometric amount of organic/inorganic reducing agents in a homogeneous system.^3^ Therefore, developing a homogeneous catalytic system for reducing POMs by hydrogen gas will provide new chemistry, such as development of hydrogen storage materials, green redox catalysts, fuel cells, and mechanistic studies of hydrogen activation.

Hydrogenases catalyze the reversible oxidation and production of hydrogen gas, wherein the electrons transferring from/to their active sites *via* iron–sulfur clusters is crucial for their metabolism.^4^ To imitate their highly-efficient catalytic activities under mild conditions, various types of hydrogenase model complexes have been synthesized to date,^5^ and we recently reported that the [NiRu] complex could catalytically convert hydrogen gas into protons and electrons through a heterolytic cleavage mechanism.^6^ Since hydrogen gas is one of the most ecofriendly reducing agents in terms of cost and atom efficiency, developing a catalytic system to extract protons and electrons from hydrogen gas by mimicking hydrogenases is of growing importance.^7^ However, utilizing extracted protons and electrons as designed is still difficult partly owing to the low catalytic efficiency and the absence of appropriate redox active mediators like iron-sulfur clusters in organisms.^8^

Herein, we focused on utilizing hydrogenase model complex for reducing POMs by hydrogen gas and for the first time reported the reduction of the α-Dawson-type POM, K_6_[P_2_W_18_^6+^O_62_] (K_6_[II_ox_]), by hydrogen gas with the hydrogenase model complex, [Ni^2+^(L)Ru^2+^(H_2_O){η^6^-C_6_(CH_3_)_6_}](NO_3_)_2_ ([I](NO_3_)_2_, L = *N*,*N*′-dimethyl-3,7-diazanonane-1,9-dithiolato),^6^ as a homogeneous catalyst in aqueous media under very mild conditions (pressure of hydrogen gas, ≤0.1 MPa; reaction temperature, 293–333 K). The kinetic study of this catalytic system revealed the small activation energy for activating hydrogen gas and reducing POMs (*E*_a_ = 51.2 kJ mol^−1^). The turnover number (TON) reached to 1975 for 6 h, showing a high-performance homogeneous catalytic system for extracting and storing protons and electrons from hydrogen gas.

## Results and discussion

**Fig. 1:**
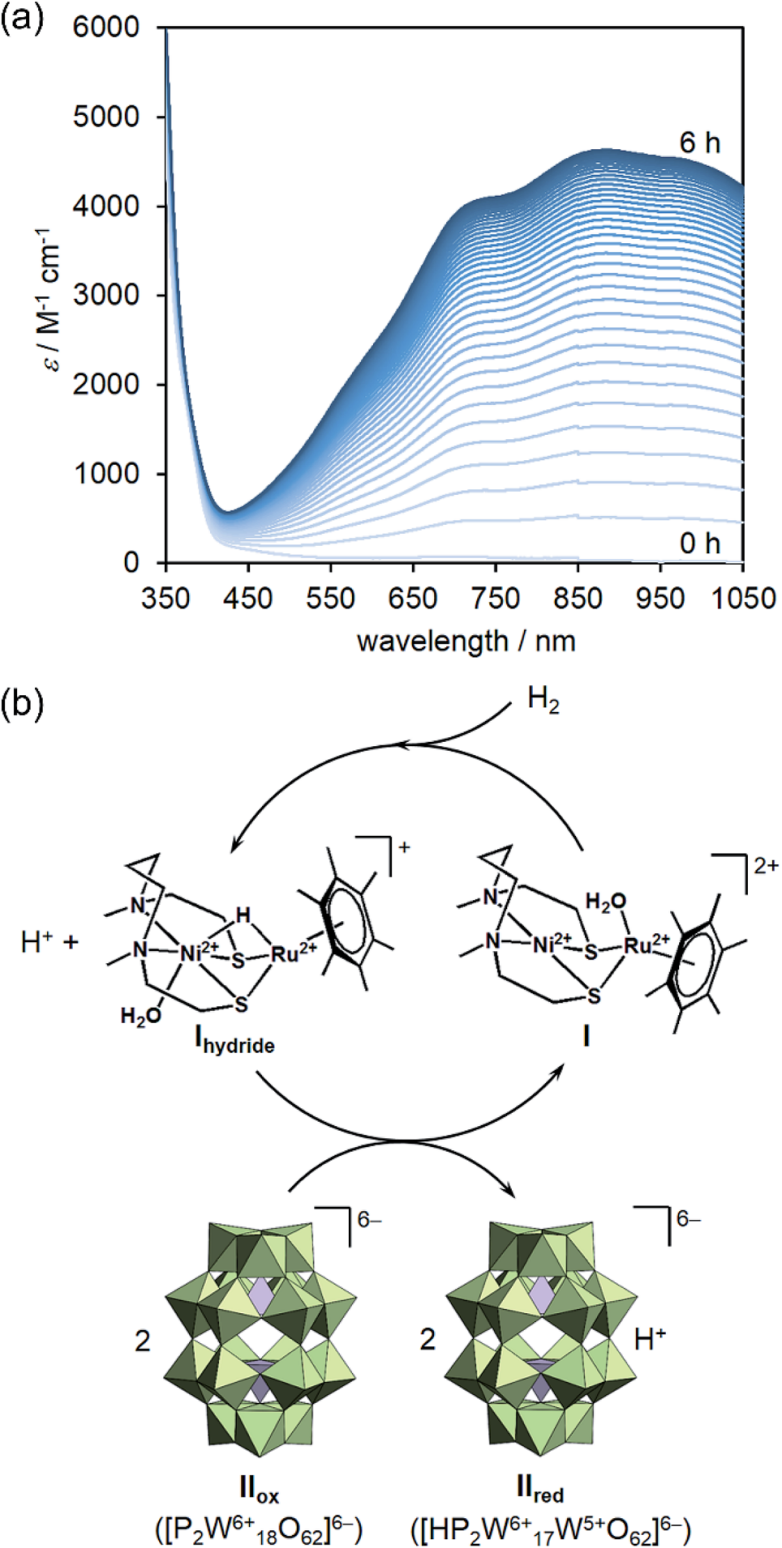
(a) UV-vis spectra of the reaction solution measured every 10 min. Reaction conditions: II_ox_ (0.5 mM), I (0.05 mM), sodium acetate buffer (pH 4, 25 mM, 3 mL), 298 K, under Ar (0.1 MPa), the reaction was initiated by bubbling hydrogen gas for 1 min. (b) Proposed catalytic mechanism for reduction of II_ox_ with I by hydrogen gas. The atoms of POMs are represented by polyhedra; [WO_6_]^6−^ and [WO_6_]^7−^: light green, [PO_4_]^3−^: gray.

**Fig. 2:**
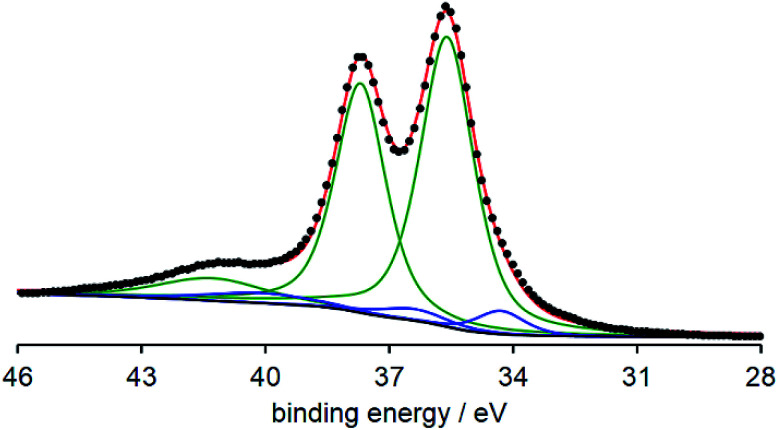
XPS spectrum of the vacuum-dried sample of the reaction solution after forming II_red_. The black dots represent the obtained spectrum. The green and blue lines represent the best fitting curves for W^6+^ and W^5+^ species, respectively, and the red line represents the sum of them.

To begin with, hydrogen gas was bubbled into the sodium acetate buffer solution (25 mM, pH 4.1) containing I (0.05 mM) and II_ox_ (0.5 mM) to investigate the catalysis of I at 298 K under Ar. After bubbling hydrogen gas for 1 min, the color of the solution in a sealed quartz cell gradually turned from pale yellow into dark blue. The UV-vis spectra of the solution showed the increase of absorption bands at around 555, 750, 878, and 995 nm assignable to the W^5+^-to-W^6+^ intervalence charge transfer (IVCT) process (*ε* = 4630 M^−1^ cm^−1^ at 878 nm, 6 h incubation after bubbling hydrogen gas for 1 min), thereby suggesting the reduction of II_ox_ (Fig. 1a). In contrast, the UV-vis spectra in the range of 550–1050 nm hardly changed in the absence of hydrogen gas, I, or II_ox_ (Fig. S1, ESI†), indicating the I-mediated reduction of II_ox_ by hydrogen gas. Since the UV-vis spectrum measured after 6 h incubation could be superimposed on that of electrochemical one-electron-reduced solution of II_ox_ (Fig. S2, ESI†), the reduced II_ox_ was proved to be a one-electron-reduced species (K_6_[HP_2_W_17_^6+^W^5+^O_62_], K_6_[II_red_]). The yield of II_red_ was calculated using the absorption coefficient at 878 nm and reached to >99% when using catalytic amount of I (10 mol%) (Fig. S3, ESI†).^9^ The X-ray photoelectron spectroscopy (XPS) spectrum of the vacuum-dried sample of the reaction solution after forming II_red_ in the W4f region was measured. The spectrum showed three major peaks for W4f_7/2_ (35.6 eV), W4f_5/2_ (37.7 eV), and W5p_3/2_ (41.3 eV) assignable to W^6+^ species together with three minor peaks for W4f_7/2_ (34.3 eV), W4f_5/2_ (36.4 eV), and W5p_3/2_ (40.1 eV) assignable to W^5+^ species with 7% area ratio, supporting the one-electron reduction of II_ox_ (Fig. 2). Therefore, based on the above-mentioned results, I could act as homogeneous catalyst to activate hydrogen gas and to give II_red_ in high yield. It is noteworthy that this system is the first example of homogeneous catalytic reduction of POMs by hydrogen gas.

**Fig. 3:**
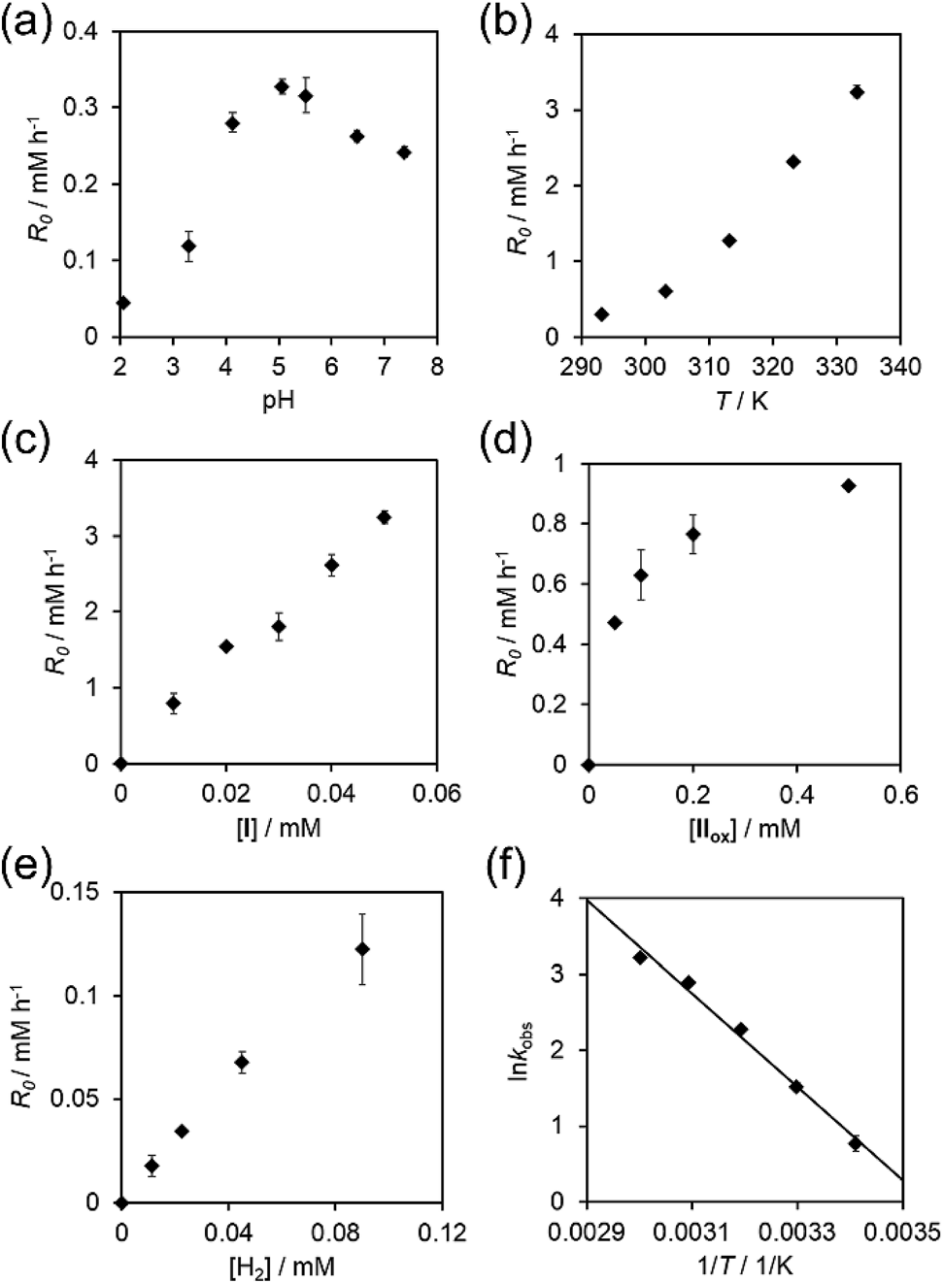
Dependences of the initial reaction rates on (a) pH of the solution, (b) the reaction temperature, (c) the concentration of I, (d) the concentration of II_ox_, and (e) the concentration of hydrogen gas. (f) Arrhenius plots for I-catalyzed reduction of II_ox_. The observed rate constants (*k*_obs_) were determined from the initial part of the reaction. Line fit: ln *k*_obs_ = 21.84−6159.3/*T*.

The initial reaction rate *R*_0_ (mM h^−1^), which was calculated by time-course UV-vis spectra at 878 nm, was dependent on pH values and reaction temperatures of buffer solutions (Fig. 3a and b). The plot of pH dependence showed that *R*_0_ increased with increasing pH values and reached to the maximum value of 3.3 × 10^−1^ mM h^−1^ at pH 5.1, and then, *R*_0_ decreased with increasing pH values above 5.1. This type of pH dependence with a maximum was also observed in the studies on the H^+^/D^+^ exchange reaction and the reduction of Cu^2+^ by hydrogen gas with I.^10^ The plot of temperature dependence showed that *R*_0_ increased with increasing reaction temperatures.

Next, the catalytic mechanism was investigated using pH 5.1 buffer solution at 333 K. To determine the active species for the reduction of II_ox_, a hydride complex of I ([Ni^2+^(H_2_O)(L)Ru^2+^(H){η^6^-C_6_(CH_3_)_6_}](NO_3_), [I_hydride_](NO_3_)), which was known to be formed by reacting I with hydrogen gas in an acidic solution,^6^ was added to a deaerated solution of II_ox_. When adding I_hydride_ (0.35 μmol) into the solution of II_ox_ (0.5 mM, 3 mL), the color of the solution immediately changed into deep blue. Since the UV-vis spectrum of the resulting solution showed that the yield of II_red_ reached to 0.63 μmol after 0.5 h incubation, 1.8 equivalents of II_ox_ with respect to I_hydride_ were reduced to II_red_ (Fig. S4, ESI†).^11^ By addition of 1 equivalent of I_hydride_ with respect to II_ox_, the UV-vis spectrum exhibited the formation of 1 equivalent of II_red_, thus indicating that two-electron reductions (I_hydride_ + II_ox_ + H^+^ → I + [H_2_P_2_W_16_^6+^W_2_^5+^O_62_]^6−^) did not occur. This result also supported the formation of one-electron-reduced species in the catalytic study (Fig. S2, ESI†). On the basis of these results and kinetics below, the reaction mechanism for I-catalyzed reduction of II_ox_ by hydrogen gas was proposed as follows (Fig. 1b): Firstly, hydrogen gas was activated by I to form I_hydride_ and a proton (eqn (1)). Then, 2 equivalents of II_ox_ were reduced by I_hydride_ using one proton, followed by the regeneration of I and the formation of II_red_ (eqn (2)).

1 **I** + H_2_ → **I**_**hydride**_ + H^+^

2 **I**_**hydride**_ + 2**II**_**ox**_ + H^+^ → **I** + 2**II**_**red**_

The kinetic study on the reduction of II_ox_ was investigated by the time-course UV-vis spectra of the reaction solutions. The first-order dependence of the initial reaction rates *R*_0_ on the concentrations of I (0–0.05 mM, Fig. 3c) and hydrogen gas (0–0.09 mM, Fig. 3e) were observed, whereas the saturation kinetics for the dependence of *R*_0_ on the concentration of II_ox_ (0–0.05 mM, Fig. 3d) was observed. From the mass balance and steady-state approximation on I_hydride_, the overall reduction rate is expressed by the following equation:3
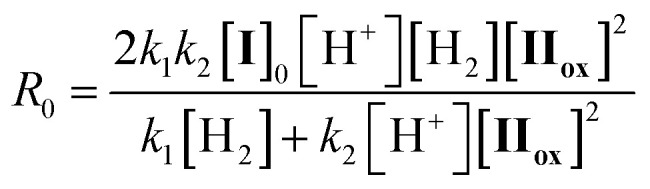
where the initial concentration of I ([I]_0_) is expressed by [I] + [I_hydride_]. On the basis of the kinetic data, the values of rate constants were calculated as follows; *k*_1_ = 1.6 × 10 s^−1^ and *k*_2_ = 6.0 × 10^8^ M^−1^ s^−1^. The dependences of the reaction rates on the concentrations of I, II_ox_, and hydrogen gas calculated by eqn (3) were approximately reproduced the experimental data (Fig. S5, ESI†). Since the reaction rate for activating hydrogen gas was much slower than that for reducing II_ox_ according to the obtained rate constants (*k*_1_[H_2_] ≪ *k*_2_[II_ox_][H^+^]), the rate-determining step was supposed to be the reaction of I with hydrogen gas to form I_hydride_ and a proton (eqn (1)), which was agreed with the result of the rapid reduction of II_ox_ by I_hydride_. The good linearity of the Arrhenius plot was observed to afford the following activation parameters: *E*_a_ = 51.2 kJ mol^−1^, ln *A* = 21.8, Δ*H*^‡^_298 K_ = 48.7 kJ mol^−1^, Δ*S*^‡^_298 K_ = −71.6 J mol^−1^ K^−1^, and Δ*G*^‡^_298 K_ = 70.1 kJ mol^−1^ (Fig. 3f). The present activation energy was much lower than free energies for the cleavage of dihydrogen in water (homolytic, 442 kJ mol^−1^; heterolytic, 143 kJ mol^−1^),^12^ showing the successful reduction of energy barrier to activate hydrogen gas by using the catalyst I. The negative value of the activation enthalpy Δ*S*^‡^_298 K_ suggested that a bimolecular transition state (hydrogen adduct of I before forming I_hydride_) was included in the rate-determining step.^13^

**Fig. 4:**
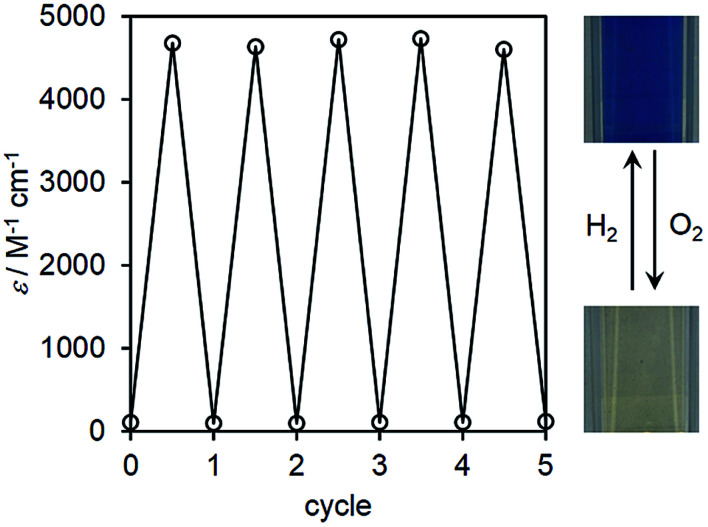
Reversible changes of the absorption coefficients observed at 878 nm. Insets: images of the reaction solutions under hydrogen and oxygen gases.

When the reaction was carried out with 0.004 mol% of I at 333 K, the yield of II_red_ reached to 79% for 6 h, resulted in a high TON of 1975, which was the highest value for the homogeneous catalytic reduction of inorganic substrates by hydrogen gas, to the best of our knowledge (Table S1†). The UV-vis spectrum of the resulting solution hardly changed in a sealed vessel for more than two weeks at room temperature, suggesting that II_ox_ could stably store protons and electrons. By addition of sodium nitrite (15 μmol, 10 equivalents with respect to II_red_) into the blue reaction solution containing II_red_ (0.5 mM, 3 mL), which was formed by I-catalyzed reduction of II_ox_ under hydrogen gas, the color of the solution changed into pale yellow, indicating the reduction of sodium nitrite and oxidation of II_red_. The conversion of II_red_ reached to 99% for 4 h (Fig. S6, ESI†), thus demonstrating the successful re-extraction of protons and electrons *via*II_ox_/II_red_ as mediators.^14^ Since II_red_ could also be oxidized by molecular oxygen, the ability to recycle this system was investigated by bubbling hydrogen gas and oxygen gas alternately. After forming II_red_ by bubbling hydrogen gas into the reaction solution containing I (0.05 mM) and II_ox_ (0.25 mM), oxygen gas was bubbled to re-oxidize II_red_. This process was repeated five times, and the yield of II_red_ in each step was monitored by measuring the UV-vis spectrum. Although the initial reaction rate gradually decreased with each cycle, II_red_ was obtained in >99% yield (Fig. 4 and S7, ESI†), indicating that this system was recyclable at least five times with the high stabilities of both the catalyst I and the mediator II_ox_.

## Conclusions

In conclusion, a homogeneous catalytic system for extracting and storing protons and electrons from hydrogen gas was developed by using a POM and a hydrogenase model complex for the first time. The present system showed the high yield of reduced POM with *ca.* 2000 TON, demonstrating a high-performance catalytic system. Extracted protons and electrons could temporary be stored in POMs and released by addition of oxidants, showing that POMs could act as mediators to transport protons and electrons. Moreover, this catalytic system was recyclable at least five times with >99% yield of reduced species. We envisage that these findings would be applied to the development of new catalytic systems and energy storage materials using hydrogen gas under mild conditions.

## Experimental section

### Materials

Hydrochloric acid (Wako), acetic acid (Wako), sodium acetate (Wako), citric acid monohydrate (Wako), disodium hydrogenphosphate (Wako), sodium dihydrogenphosphate dihydrate (Wako), sodium hydroxide (Wako), sodium nitrite (Wako), chloroform (Wako), and 1,1′-dibenzyl-4,4′-bipyridinium dichloride hydrate (TCI) were purchased and used as received. Compounds [Ni^2+^(L)Ru^2+^(H_2_O){η^6^-C_6_(CH_3_)_6_}](NO_3_)_2_ ([I](NO_3_)_2_, L = *N*,*N*′-dimethyl-3,7-diazanonane-1,9-dithiolato), [Ni^2+^(H_2_O)(L)Ru^2+^(H){η^6^-C_6_(CH_3_)_6_}](NO_3_) ([I_hydride_](NO_3_)), and K_6_[P_2_W_18_^6+^O_62_] (K_6_[II_ox_]) were synthesized according to the reported procedures.^6,15^ The buffer solutions were prepared using hydrochloric acid (pH 2.06), citric acid/sodium citrate (pH 3.30), acetic acid/sodium acetate (pH 4.13, 5.07, 5.52), or phosphorus acid/sodium phosphate (pH 6.48, 7.38).

### Instruments

UV-vis spectra were measured on JASCO V-670. IR spectra were measured on PerkinElmer Spectrum Two. The pH values of the buffer solutions were determined using TOA DK MH-30R pH meter.

### Controlled potential electrolysis

The controlled potential electrolysis of II_ox_ (2 mM) in acetate buffer (*ca.* 60 mL, pH 4, 25 mM) was carried out using an electrolyzer separated by glass frit. Pt electrodes were used as cathode and anode, which were connected to a BAS electrochemical analyzer 600D. Nitrogen gas was bubbled into the solution during the electrolysis with stirring. The solutions of one- and two-electron reduced II_ox_ were prepared by the electrolysis at −0.1 and −0.27 V *vs.* Ag/AgCl, respectively.

### XPS analysis

The XPS analysis was performed using a ULVAC-PHI PHI 5000 VersaProbe II under Al Kα radiation (*hν* = 1486.6 eV, 15 kV, 25 W). The peak positions were calibrated by the W4f_7/2_ (35.60 eV) of W^6+^ atoms in POMs, and the baseline was subtracted by the Shirley method. The curve fitting was performed with the spin–orbit separation Δ*E*_P_(W4f_5/2_–W4f_7/2_) of 2.1 eV and the intensity ratio *I*(W4f_5/2_)/*I*(W4f_7/2_) of 0.75.^16^ The ratio of Lorentzian to Gaussian varied in the range of 50 ± 5%. The sample was prepared as follows: hydrogen gas was bubbled into the aqueous solution (20 mL) containing I (0.05 mM) and II_ox_ (0.25 mM) for 10 min. The UV-vis spectrum of the resulting solution was measured after *ca.* 1 h incubation at 323 K, showing that the yield of II_red_ reached to >99%. The resulting solution was dried *in vacuo* to give a dark blue powder, which was used for the measurement.

### Procedures for catalytic reduction of II_ox_

The buffer solution of I was added to the buffer solution of II_ox_ to give a pale-yellow reaction solution, followed by bubbling Ar for 10 min. The reaction was initiated by bubbling H_2_ or adding H_2_-containing aqueous solution into the reaction solution in a sealed quartz cell. In a separate experiment, the concentration of H_2_ in water was determined by measuring the intensity of absorption band at 600 nm for one-electron reduced 1,1′-dibenzyl-4,4′-bipyridinium dichloride (*ε* = 7.4 × 10^3^ M^−1^ cm^−1^) which was formed by the reaction of 1,1′-dibenzyl-4,4′-bipyridinium dichloride with H_2_ using Pt as a catalyst. The catalyst I after using catalytic reaction was obtained by the following procedure: Ar was bubbled for 1 h into the reaction solution (100 mL, pH 5, 25 mM) containing I (0.5 mM) and II_ox_ (5 mM), followed by bubbling hydrogen gas for 10 min. The UV-vis spectrum of the resulting solution after 16 h incubation showed that II_ox_ was completely reduced to II_red_. Chloroform (100 mL) was added to the resulting solution, and then, the mixture was shaken vigorously to give orange precipitates, which was collected by filtration and measured by the IR spectroscopy. The recycle experiment in a homogeneous system was performed at 333 K by the following procedure: Ar was bubbled for 5 min into the reaction solution (3 mL, pH 5, 25 mM) containing I (0.05 mM) and II_ox_ (0.25 mM), followed by bubbling hydrogen gas for 3 min. The UV-vis spectrum of the resulting solution was measured to determine the yield of II_red_ after 15–90 min incubation. Then, oxygen gas was bubbled into the resulting solution for 3 min to give a colorless solution after 15–45 min incubation. The UV-vis spectrum of the resulting solution was measured to confirm that II_red_ was completely re-oxidized to II_ox_. These processes were repeated five times against the same reaction solution.

## Conflicts of interest

There are no conflicts to declare.

## Supplementary Material

RA-009-C9RA04396A-s001
