# Supplementary material for: Homogeneous catalytic reduction of polyoxometalate by hydrogen gas with a hydrogenase model complex
Source: RSC Adv. 2019 Jun 21;9(34):19518–22. doi: 10.1039/c9ra04396a (PMC9065399; doi:10.1039/c9ra04396a)
Supplement: RA-009-C9RA04396A-s001 [file RA-009-C9RA04396A-s001.pdf]

Electronic Supplementary Material (ESI) for RSC Advances.
This journal is © The Royal Society of Chemistry 2019

Electro Supplementary Information (ESI) for

Homogeneous catalytic reduction of polyoxometalate by hydrogen gas with hydrogenase model complex

Takuo Minato,*[a,b] Takahiro Matsumoto [a,b,c] and Seiji Ogo *[a,b,c]

[a] Department of Chemistry and Biochemistry, Graduate School of Engineering, Kyushu University

[b] International Institute for Carbon-Neutral Energy Research (WPI-I2CNER), Kyushu University

[c] Centre for Small Molecule Energy, Kyushu University

Kinetic Derivation: Dihydrogen was activated by **I** to form $\mathbf{I_{hydride}}$ and a proton (eqn (S1)).

$$I + H_2 \rightarrow I_{hydride} + H^+ \qquad (S1)$$

Then, 2 equivalents of $\mathbf{II_{ox}}$ were reduced by $\mathbf{I_{hydride}}$ using one proton, followed by the regeneration of **I** and the formation of $\mathbf{II_{red}}$ (eqn (S2)).

$$I_{hydride} + 2II_{ox} + H^+ \rightarrow I + 2II_{red} \qquad (S2)$$

From the mass balance and the steady-state approximation on $\mathbf{I_{hydride}}$ (eqn (S3) and eqn (S4)), the rate of the formation of $\mathbf{II_{red}}$ could be expressed by eqn (S5).

$$[I]_0 = [I] + [I_{hydride}] \qquad (S3)$$

$$\frac{d[I_{hydride}]}{dt} = k_1[I][H_2] + k_2[I_{hydride}][II_{ox}]^2[H^+] = 0 \qquad (S4)$$

$$R_0 = \frac{d[II_{red}]}{dt} = \frac{2k_1k_2[I]_0[H^+][H_2][II_{ox}]^2}{k_1[H_2] + k_2[H^+][II_{ox}]^2} \qquad (S5)$$

From eqn (S5), eqn (S6) and eqn (S7) were obtained.

$$\frac{1}{R_0} = \left(\frac{1}{2k_1[I]_0}\right)\frac{1}{[H_2]} + \frac{1}{2k_2[I]_0[H^+][II_{ox}]^2} \qquad (S6)$$

$$\frac{1}{R_0} = \left(\frac{1}{2k_2[I]_0[H^+]}\right)\frac{1}{[II_{ox}]^2} + \frac{1}{2k_1[I]_0[H_2]} \qquad (S7)$$

Plots of $1/R_0$ versus $1/[H_2]$ and $1/R_0$ versus $1/[\mathbf{II_{ox}}]$ were shown in Fig. S12 and Fig. S13. The values of k_1 and k_2 were determined from these line fits as follows: $k_1 = 1.6 \times 10$ s^{-1} and $k_2 = 6.0 \times 10^8$ M^{-1} s^{-1}. The experimental data in Fig. S5 could be fitted with simulation curves using these rate constants and eqn (S5).

Table S1. TON for the catalytic reduction of inorganic compounds by hydrogen gas in homogeneous system.

Entry	Catalyst	Electron acceptor	Reaction conditions					TON	Reference
			Pressure of H_2	Temperature	Amount of catalyst	Solvent	Base		
1	**I**	$\mathbf{II}_{\mathbf{ox}}$	0.1 MPa	333 K	0.004 mol%	sodium acetate buffer	–	1975	This work
2	$Cp^{C6F5}Fe(P^{tBu}{}_2N^{Bn}{}_2)H]$	$[Cp_2Fe]^+$	0.1 MPa	295 K	2 mol%	PhF	*N*-methylpyrrolidine or Et_3N	25	S1
3	**I**	Cu^{2+}	0.8 MPa	323 K	0.5 mol%	sodium acetate buffer	–	17	S2
4	$Cp^*Ir(^tBA^FPh)$	Ag^+	0.04 MPa	296 K	16.7 mol%	THF	2,6-(*t*-Bu)$_2C_5H_3N$	6	S3
5	$PdCl_2$	Fe^{3+}	0.06 MPa	353 K	85 mol%	3 M HCl	–	1.2[a]	S4

[a] calculated by using provided data.

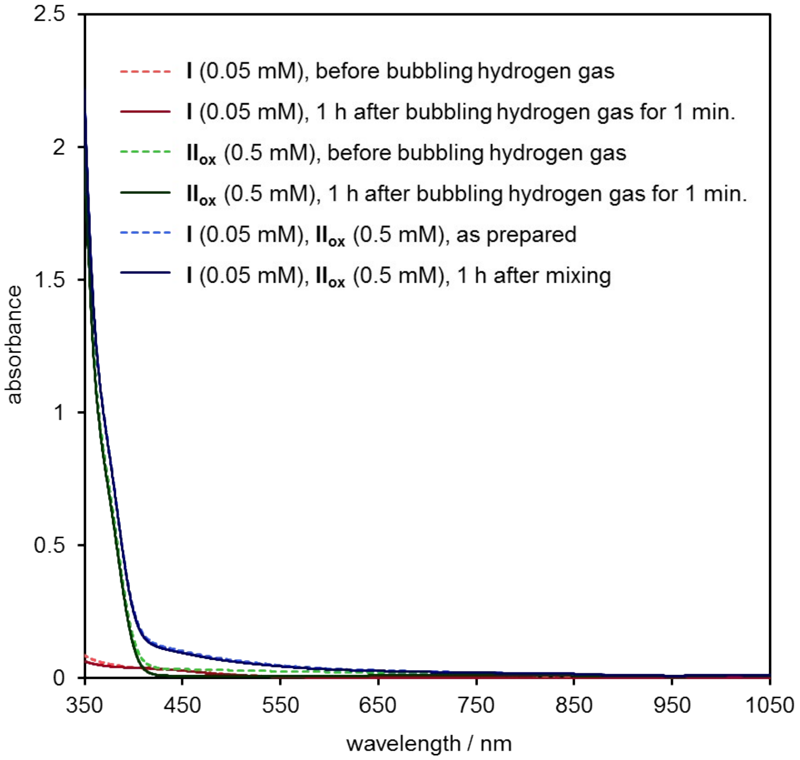

Fig. S1 UV-vis spectra of reaction solutions in the absence of **II**$_{\mathbf{ox}}$ (red), **I** (green), or hydrogen gas (blue).

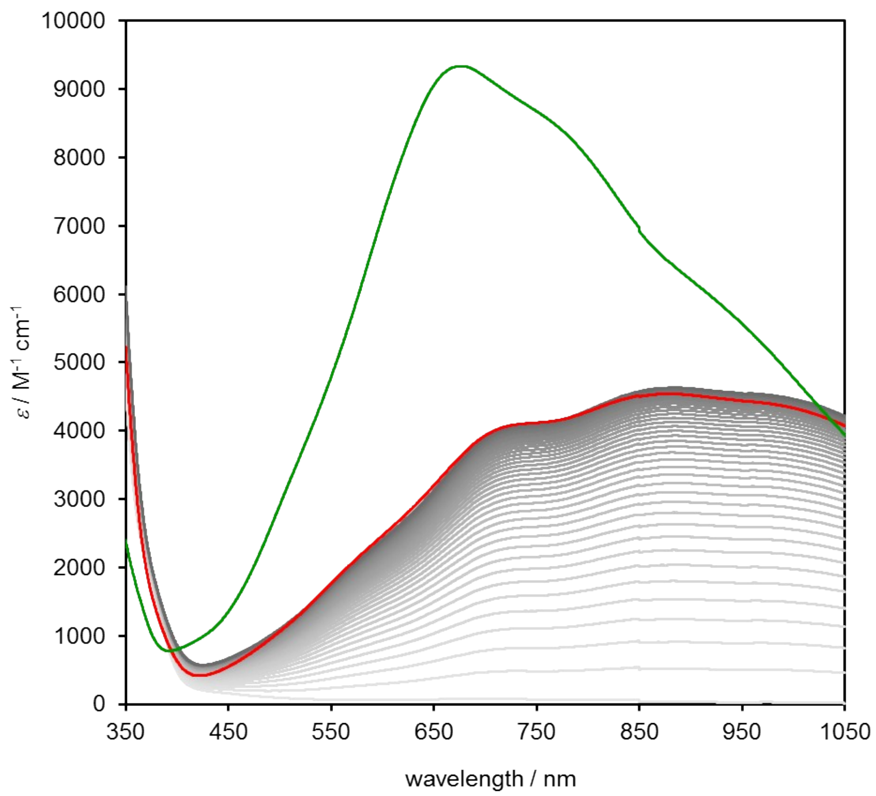

Fig. S2 UV-vis spectra of electrochemically reduced $\mathbf{II}_{\mathbf{ox}}$. Red and green spectra represent one-electron and two-electron reduced $\mathbf{II}_{\mathbf{ox}}$, respectively. Gray spectra represent the same spectra as shown in Fig. 1a. Reaction conditions: $\mathbf{II}_{\mathbf{ox}}$ (0.5 mM), sodium acetate buffer (pH 4.1, 25 mM, ca. 60 mL), 298 K, under N_2 (0.1 MPa). The bulk electrolysis was carried out at –0.10 and –0.27 V for one-electron and two-electron reduction, respectively.

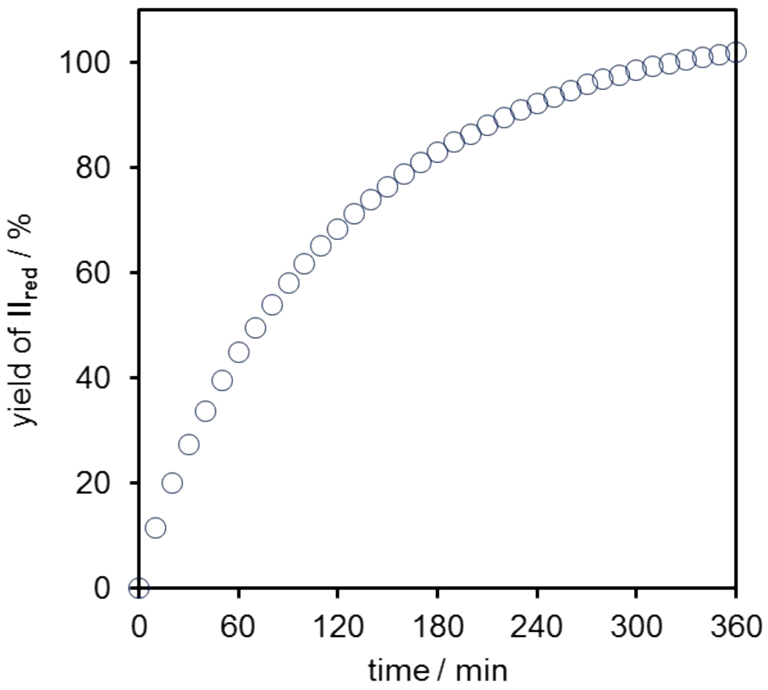

Fig. S3 Reaction profile for **I**-catalyzed reduction of $\mathbf{II_{ox}}$. Reaction conditions: $\mathbf{II_{ox}}$ (5 mM), **I** (0.5 mM), sodium acetate buffer (pH 4.1, 25 mM, 3 mL), 293 K, under Ar (0.1 MPa). The reaction was initiated by bubbling H_2 for 1 min. Yield of $\mathbf{II_{red}}$ was determined by time-course UV-vis spectra at 878 nm.

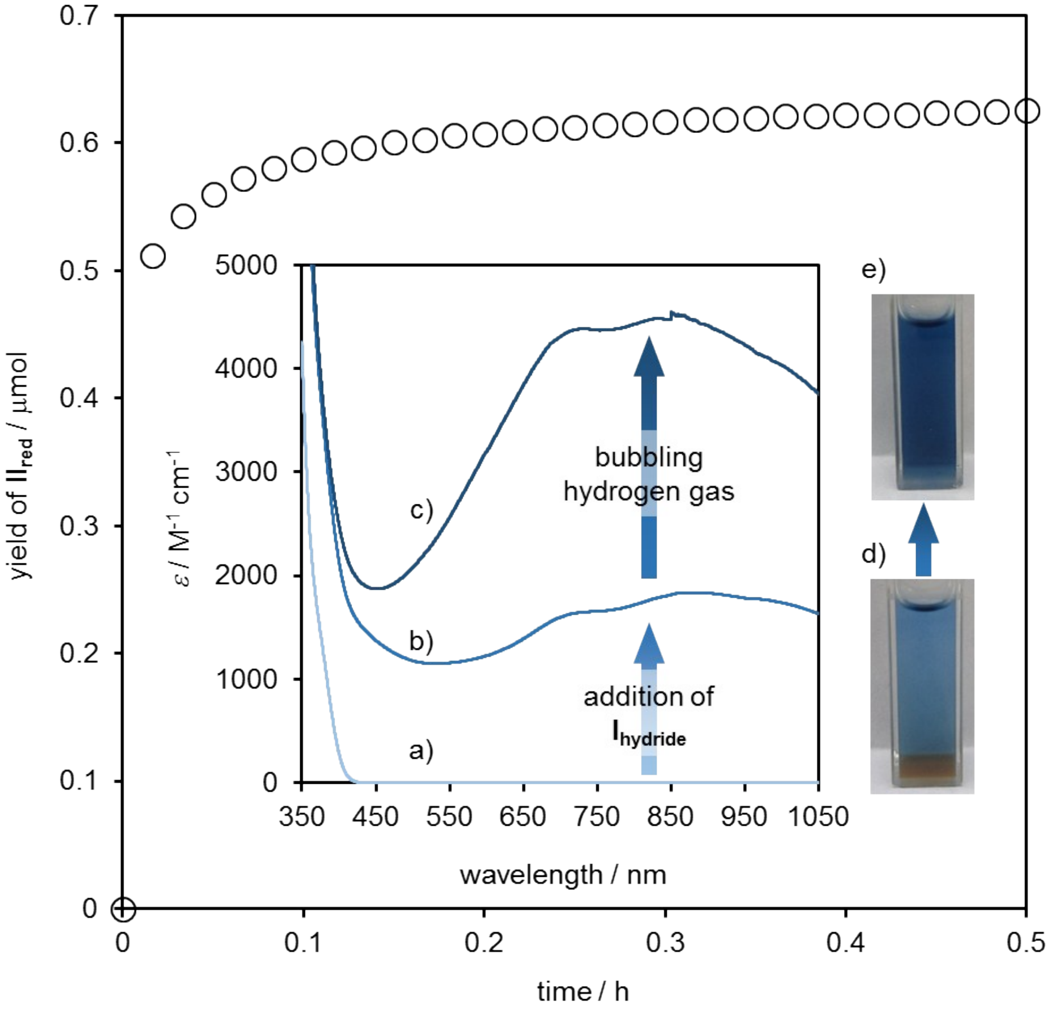

Fig. S4 Reaction profile for reduction of $\mathbf{II}_{\mathbf{ox}}$ by $\mathbf{I}_{\mathbf{hydride}}$. Reaction conditions: $\mathbf{II}_{\mathbf{ox}}$ (1.5 µmol), $\mathbf{I}_{\mathbf{hydride}}$ (0.35 µmol), sodium acetate buffer (pH 5.1, 25 mM, 3 mL), 333 K, under Ar (0.1 MPa). Yield of $\mathbf{II}_{\mathbf{red}}$ was determined by time-course UV-vis spectra at 878 nm. Insets: UV-vis spectra of a) before and b) after addition of $\mathbf{I}_{\mathbf{hydride}}$, and c) 10 min after bubbling hydrogen gas for 1 min into the resulting solution. Photographs d) and e) correspond to the solutions for b) and c), respectively.

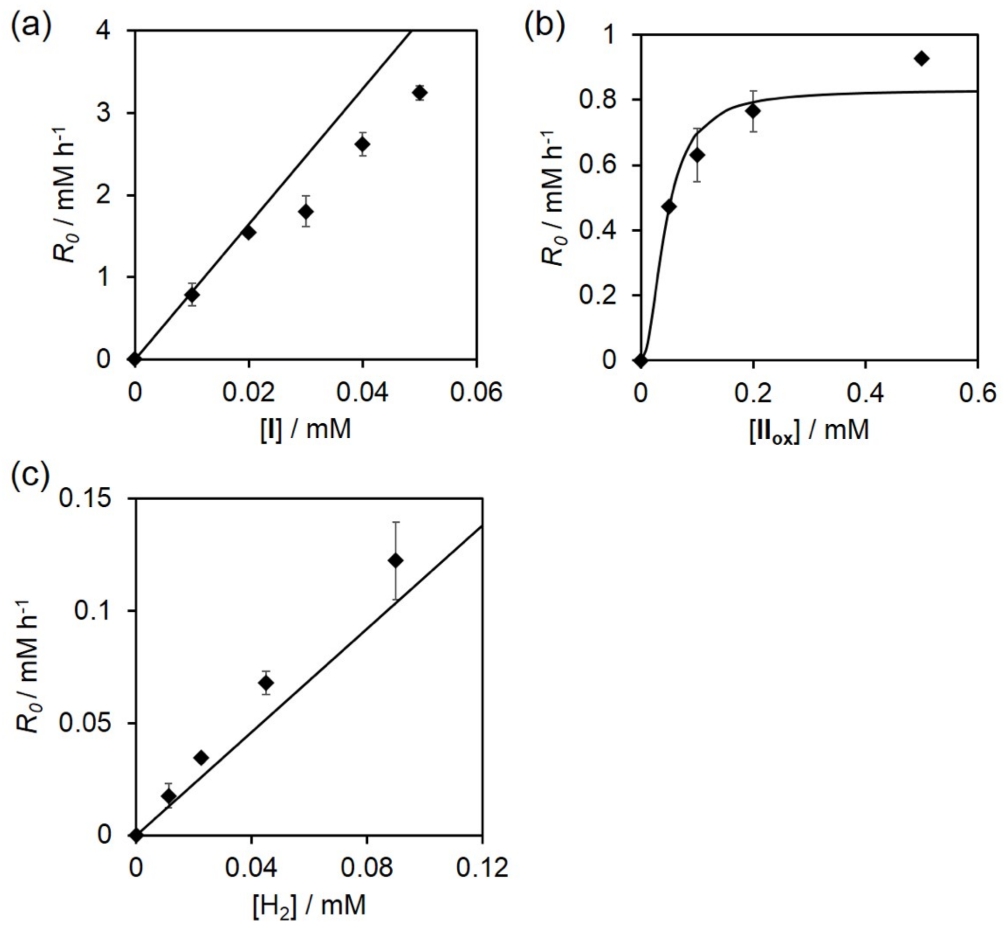

Fig. S5 Dependences of the initial reaction rates on (a) the concentration of **I**, (b) the concentration of $\mathbf{II_{ox}}$, and (c) the concentration of hydrogen. Solid lines represent simulation curves using eqn (3). The slight differences between observed initial reaction rates and the solid lines were presumably derived from the error of the concentration of hydrogen gas in reaction solutions due to its low solubility and/or the numbers of experimental points that were used for plotting the lines to calculate initial reaction rates.

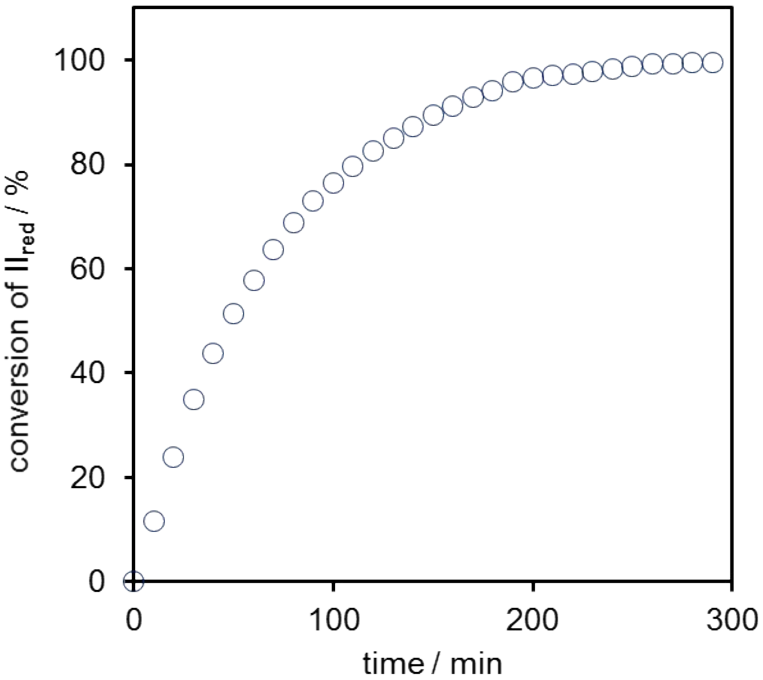

Fig. S6 Reaction profile for re-oxidation of $\mathbf{II}_{red}$ by sodium nitrite. Reaction conditions: $\mathbf{II}_{red}$ (0.5 mM), sodium nitrite (15 μmol), sodium acetate buffer (pH 5.1, 25 mM, 3 mL), 293 K, under Ar (0.1 MPa). Conversion of $\mathbf{II}_{red}$ was determined by time-course UV-vis spectra at 878 nm.

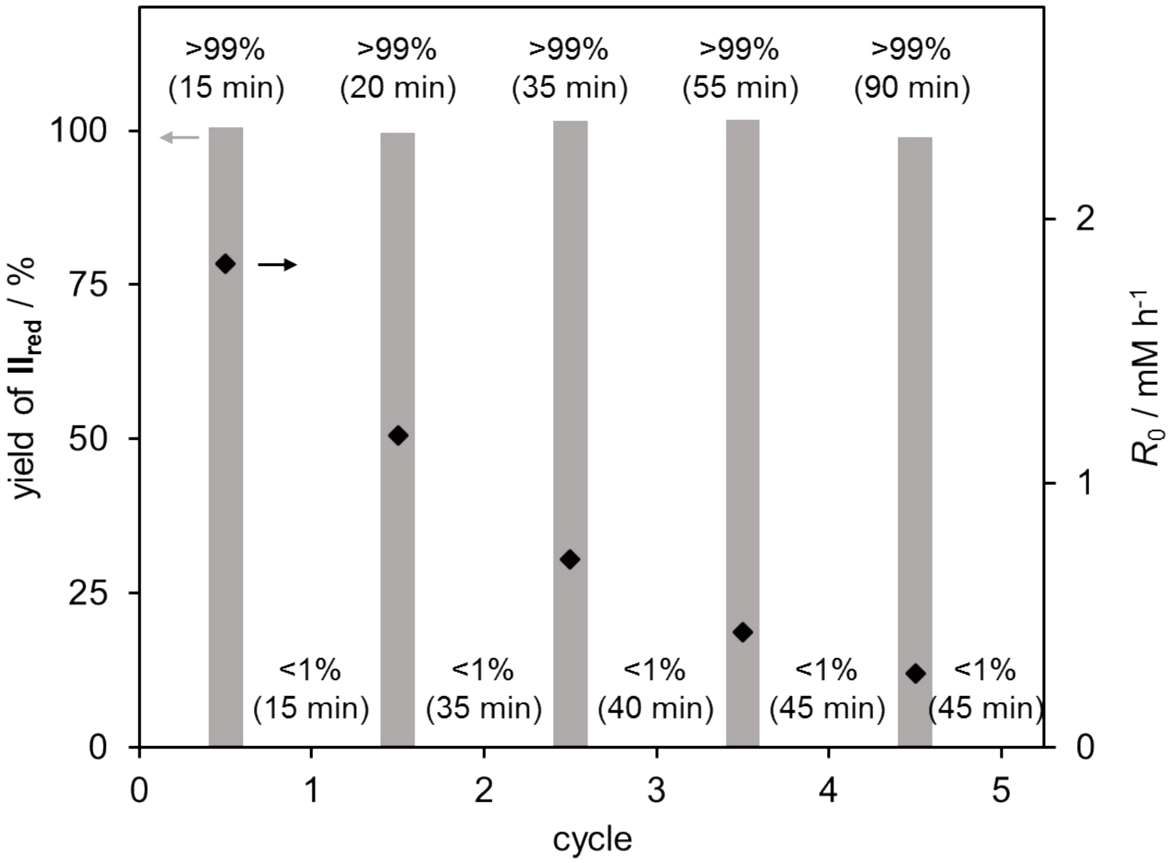

Fig. S7 Yield of $\mathbf{II}_{\mathbf{red}}$ in each step and the initial reaction rates after bubbling hydrogen gas for the recycle study.

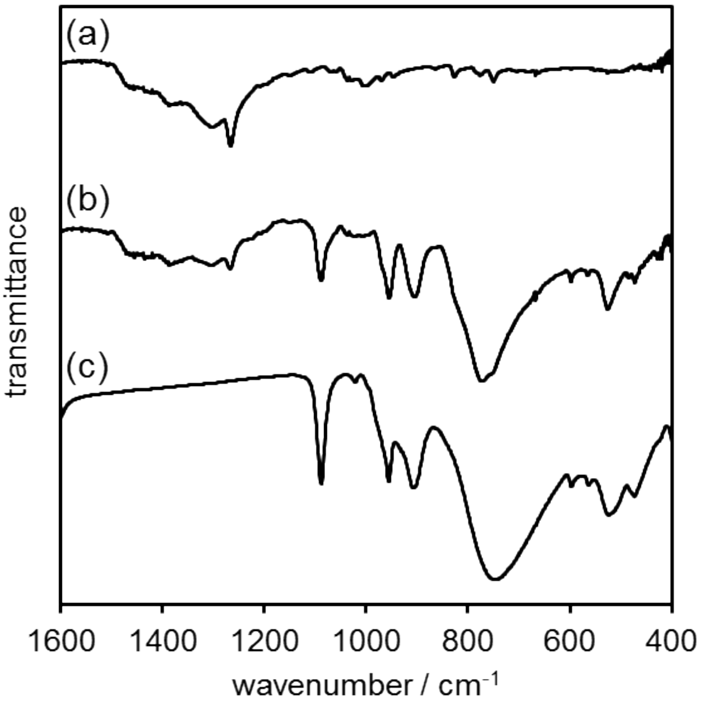

Fig. S8 IR spectra of (a) **I**, (b) precipitates obtained by addition of chloroform into the reaction solution, and (c) $\mathbf{II}_{\mathbf{ox}}$.

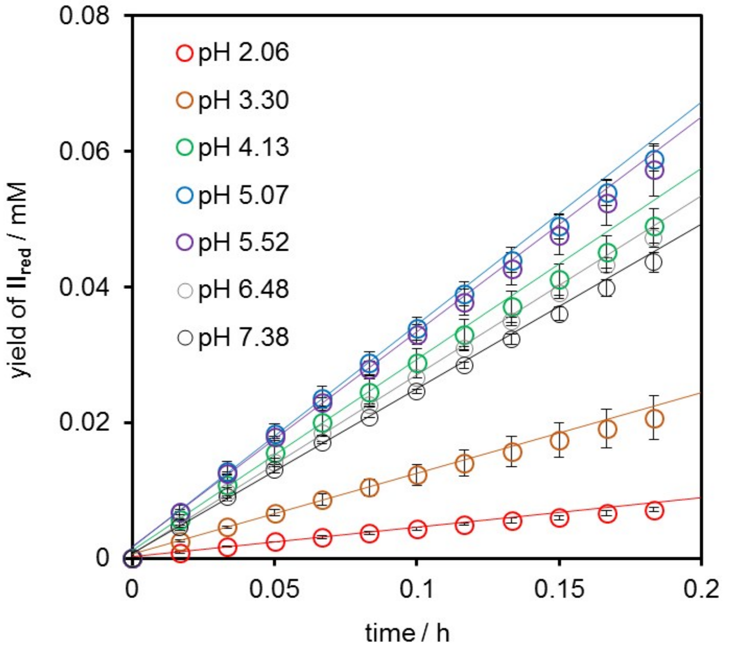

Fig. S9 Reaction profile for pH dependences of the initial reaction rate. Reaction conditions: $\mathbf{II}_{\mathbf{ox}}$ (0.5 mM), **I** (0.05 mM), buffer solutions (pH 2.1–7.4, 25 mM, 3 mL), 333 K, under Ar (0.1 MPa), the reaction was initiated by bubbling hydrogen gas for 1 min.

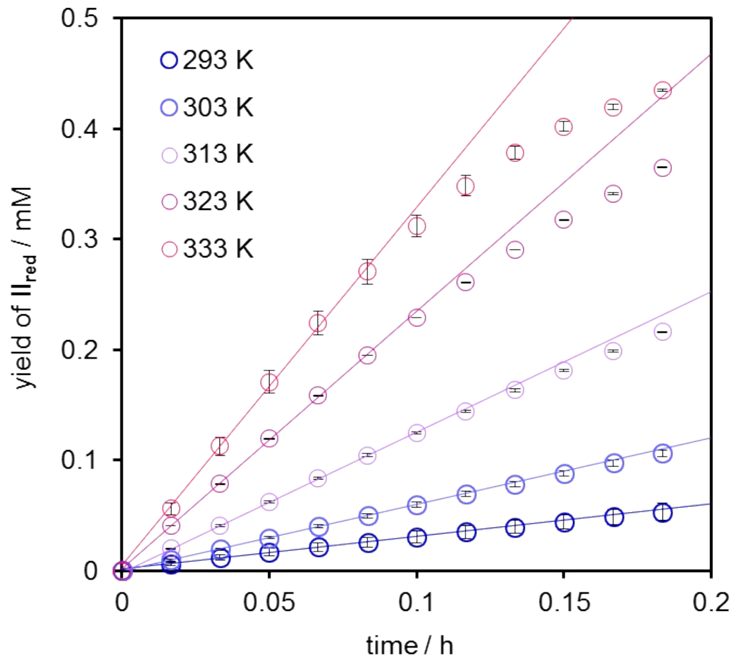

Fig. S10 Reaction profile for temperature dependences of the initial reaction rate. Reaction conditions: $\mathbf{II}_{\mathbf{ox}}$ (0.5 mM), **I** (0.05 mM), sodium acetate buffer (pH 5, 25 mM, 3 mL), 293–333 K, under Ar (0.1 MPa), the reaction was initiated by bubbling hydrogen gas for 1 min.

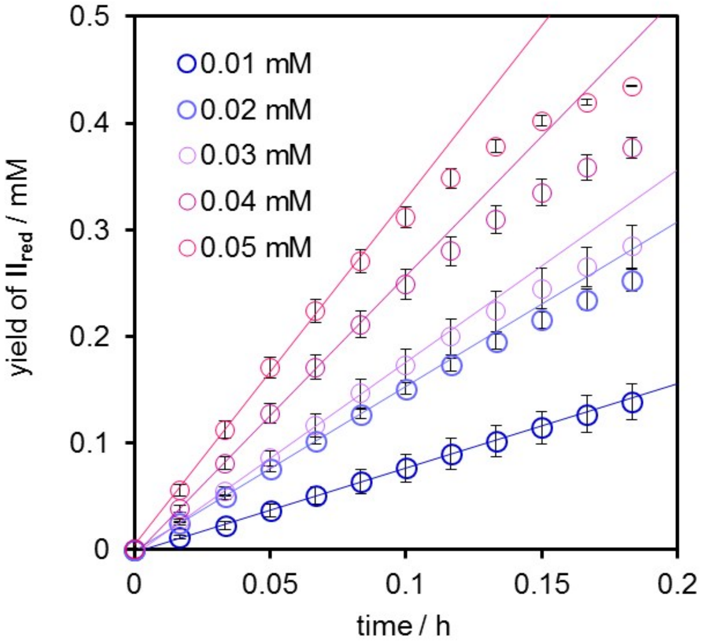

Fig. S11 Reaction profile for **I** dependences of the initial reaction rate. Reaction conditions: $\mathbf{II_{ox}}$ (0.5 mM), **I** (0–0.05 mM), sodium acetate buffer (pH 5, 25 mM, 3 mL), 333 K, under Ar (0.1 MPa), the reaction was initiated by bubbling hydrogen gas for 1 min.

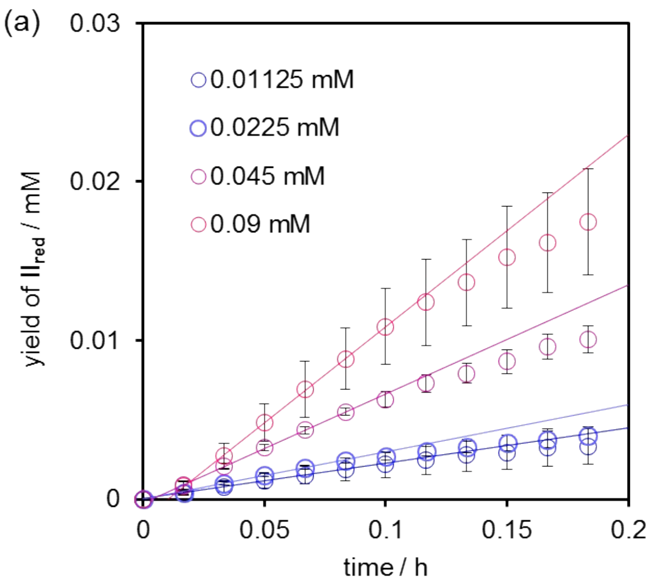

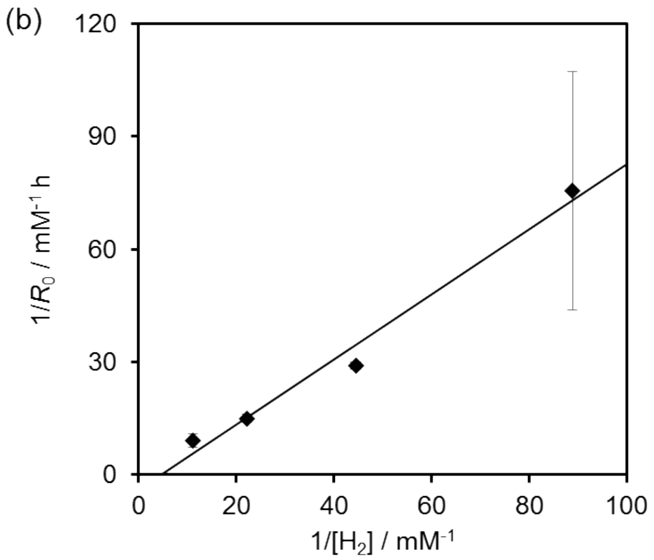

Fig. S12 (a) Reaction profile for H_2 dependences of the initial reaction rate. (b) Plot of $1/R_0$ versus $1/[H_2]$. Reaction conditions: $\mathbf{II}_{ox}$ (2 mM), **I** (0.01 mM), sodium acetate buffer (pH 5, 25 mM, 3 mL), 333 K, under Ar (0.1 MPa), the reaction was initiated by adding H_2 saturated solution.

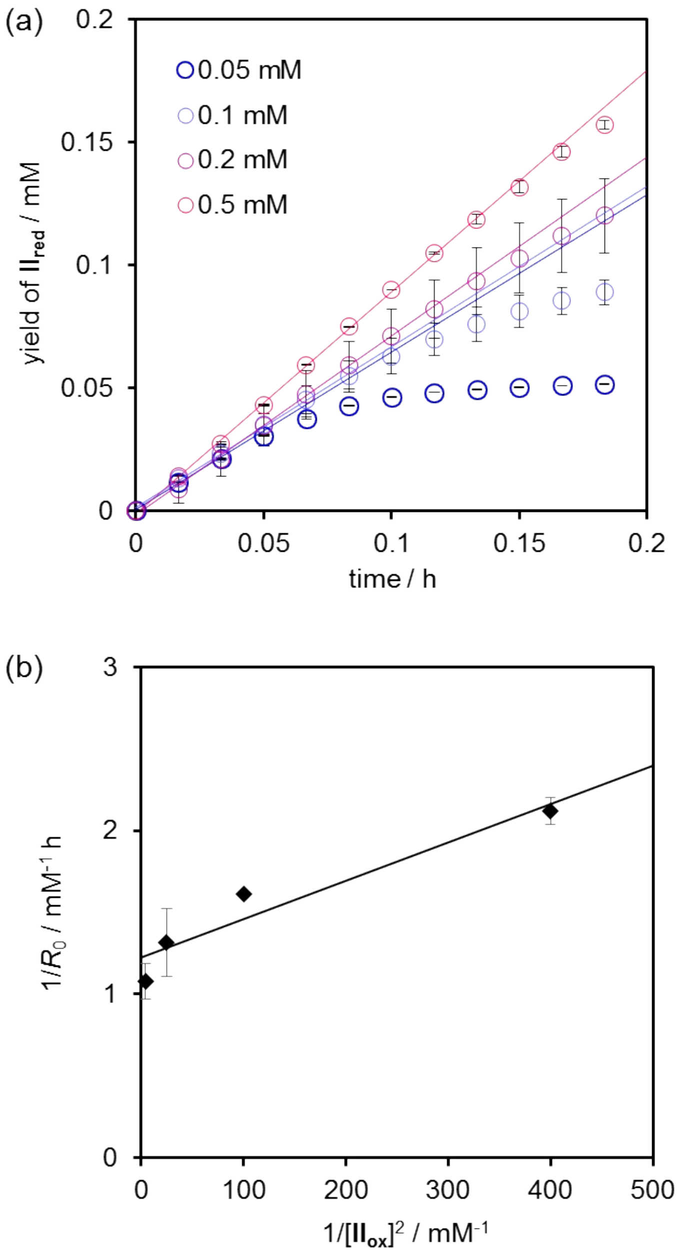

Fig. S13 (a) Reaction profile for $\mathbf{II}_{\mathbf{ox}}$ dependences of the initial reaction rate. (b) Plot of $1/R_0$ versus $1/[\mathbf{II}_{\mathbf{ox}}]^2$. Reaction conditions: $\mathbf{II}_{\mathbf{ox}}$ (0–0.5 mM), **I** (0.01 mM), sodium acetate buffer (pH 5, 25 mM, 3 mL), 333 K, under Ar (0.1 MPa), the reaction was initiated by bubbling hydrogen gas for 1 min.

References

S1 T. Liu, D. L. DuBois and R. M. Bullock, *Nat. Chem.,* 2013, ***5***, 228.

S2 T. Matsumoto, B. Kure, and S. Ogo, *Chem. Lett.,* 2008, ***37***, 970.

S3 M. R. Ringenberg, S. L. Kokatam, Z. M. Heiden and T. B. Rauchfuss, *J. Am. Chem. Soc.,* 2007, ***130***, 788.

S4 J. Halpern, J. F. Harrod and P. E. Potter, *Can. J. Chem.,* 1959, ***37***, 1446.
